# Structurally colored semitransparent perovskite solar cells using one-step deposition of self-ordering microgel particles†

**DOI:** 10.1039/d4ra00324a

**Published:** 2024-02-19

**Authors:** Osama M. Alkhudhari, Ran Wang, Zhenyu Jia, Nigel W. Hodson, Amal Alruwaili, Amal Altujjar, Eugenio Picheo, Brian R. Saunders

**Affiliations:** a Department of Materials, University of Manchester Engineering Building A Manchester M1 7HL UK brian.saunders@manchester.ac.uk osama.alkhudhari@postgrad.manchester.ac.uk; b BioAFM Facility, Faculty of Biology, Medicine and Health, University of Manchester Stopford Building, Oxford Road Manchester M13 9PT UK; c Basic Science Department, Deanship of Preparatory Year and Supporting Studies, Imam Abdulrahman Bin Faisal University Dammam 34221 Kingdom of Saudi Arabia

## Abstract

Semitransparent perovskite solar cells (STPSCs) have excellent potential for widespread application as building integrated photovoltaics. Widespread application of STPSCs could result in decreased CO_2_ footprints for buildings. Unfortunately, STPSCs tend to have poor aesthetic qualities (being usually red-brown in color) and low stability. Building on our previous work, here we use new poly(*N*-isopropylacrylamide) microgels (PNP MGs) to provide highly ordered non-close packed arrays within perovskite films that reflect some of the incident light to provide structural color to STPSCs. (MGs are swellable crosslinked polymer colloid particles.) We introduce PNP MGs into two different perovskites and achieve a wide gamut of reflected color and iridescence from the perovskite films. Devices containing the MGs have average visible transparency (AVT) values of greater than 25%. The best PCE for a MG-containing STPSC is 10.60% compared to 9.14% for the MG-free control. The MGs not only introduce structural color to the STPSCs but increase the PCE and stability. Equations are provided that enable the reflected color to be predicted from the formulation used to deposit the films. Our work shows that the self-ordering tendency of PNP MGs gives a viable new method for introducing structural color into STPSCs. Because our one-step method for introducing structural color into STPSCs is general, does not introduce any additional processing steps and is scalable whilst also improving device stability, this study may bring deployment of STPSCs closer.

## Abstract 2

Colorful semitransparent perovskite films and devices are fabricated employing self-ordering microgel particles resulting in improved power conversion efficiency and stability.

## Graphical abstract

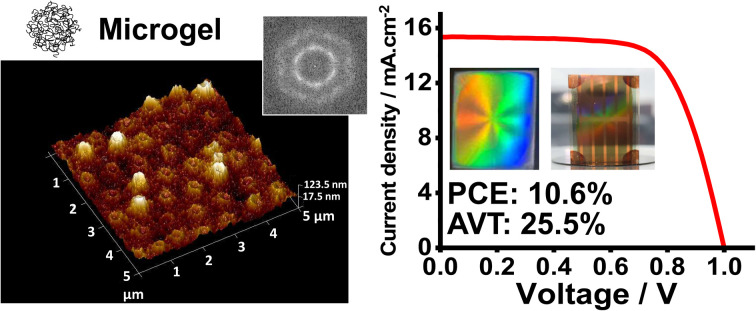

## Introduction

Perovskite solar cells (PSCs) continue to captivate the solar community because of their desirable optoelectronic properties, such as high absorption coefficient, large charge carrier diffusion length, ease of band gap tunability and facile preparation using abundant, low-cost materials.^1–5^ Since 2009, the power conversion efficiency (PCE) of PSCs has skyrocketed from 3.8%^6^ to 26.1%.^7^ Similarly, semitransparent PSCs (STPSCs) have also received considerable attention.^8–11^ They have very good potential for application in building-integrated photovoltaics (BIPV)^12,13^ such as use in windows or facades. In 2018, residential and commercial sector buildings accounted for 40% of total energy consumption in the US.^9^ Furthermore, the global BIPV market is projected to approach US $60B by 2028 with a compound growth rate of 20%.^14^ Human emotions can be highly affected by colors^15–17^ and consequently the need for producing colorful STPSCs has become increasingly important.^18^ In the present work, we address this need using microgels (MGs). MGs are crosslinked polymer colloids that swell in a good solvent.^19^ Poly(*N*-isopropylacrylamide) (PNP) MGs can form non-close-packed ordered arrays spontaneously when deposited from water as first reported by Pelton and Chibante.^20^ Such arrays can provide iridescent colors *via* diffraction.^21–23^ The present work aimed to demonstrate that structurally colored STPSCs could be produced by including PNP MGs in the perovskite precursor solution. We test the following hypotheses in this work: (1) spontaneous non-close packed hexagonal ordering of PNP MGs in a perovskite matrix can give structurally colored STPSCs. (2) Structural color from such STPSCs will be tuneable *via* control of MG-to-MG distance.

One of the most seminal STPSC papers was reported by the Oxford group whereby neutral colored STPSCs were established using perovskite micro-islands.^24^ Nanopillar-structured STPSCs have also been reported.^25^ Ag nanogrid electrodes have been used to provide STPSCs with an average visible transmittance (AVT) and PCE of 25.2% and 12.7%, respectively.^26^ Top electrode engineering resulted in a record STPSC PCE of 17.90%.^27^ A minimum AVT of 25% is generally required for STPSC application.^28^ Excellent reviews that include comprehensive surveys of STPSC AVT and PCE values have recently been published.^9,29^ Colloidal-based additives have also been used to prepare STPSCs. Sacrificial polymer colloidal monolayer templates were used by Hörantner *et al.*^30^ to construct honeycomb scaffolds for STPSCs. Zhang *et al.* developed STPSCs using a sacrificial colloid templating approach with plasma etching.^31^ Fabrication procedures for STPSCs that involve multiple processing steps and/or sacrificial colloid layers may not be easy to scale up. In contrast, here we leverage the unique tendency of PNP MGs to self-order into non-close packed arrays to achieve structurally colored STPSCs without introducing additional processing steps.

Colorful solar cells or semitransparent solar cells have enhanced potential for BIPV,^32^ wearable electronics^33^ and automotive integration.^34^ Unfortunately, conventional STPSCs tend to be red-brown in color.^35^ Color has been introduced into STPSCs by tuning the perovskite band gap,^36^ using dyes or pigments, and introducing nanostructures with engineered optical properties.^33^ Eperon *et al.* tinted their neutral colored STPSCs using dye added to the hole transport layer (HTL).^24^ Guo *et al.* added pigments to the Ag electrode to achieve colored STPSCs^37^ with an AVT of 30.2% and a PCE of 9.04%. Structural color is often used in nature^38^ and provides long term color stability. Wang *et al.* constructed bifacial color tunable STPSCs by tuning the ITO or CuSCN layer thickness.^39^ Lee *et al.* used a swelling-induced crack propagation approach to fabricate colored PSCs exhibiting a PCE of 20.1%.^40^ Yue *et al.* fabricated colored STPSCs of FAPbBr_3_ perovskite using a one-step printing blade-coated technique and employed an *in situ* intermediate phase-transition assisted approach. Their devices exhibited a PCE of 8.6% and an AVT of 45%.^41^ Here, we mix array-forming MG additives with the perovskite precursor solution to provide structurally colorful STPSCs in one step. Because the MGs are easily prepared and our approach simply applies them as an additive and does not employ bespoke charge transport layers or electrodes, we believe this study provides a uniquely facile method for preparing structurally colored STPSCs.

In this study, we use PNP MGs as 2D array-forming colloid additives in structurally colored STPSCs. PNP MGs have low size polydispersity, are surface active and can spontaneously form hexagonal 2D non-close-packed arrays when deposited on substrates. Such PNP arrays can be colorful.^21,22^ This ordered structure formation underpins the present study and is a spontaneous self-ordering transfer process. PNP MGs preferentially adsorb at the air/liquid interface in the swollen state and form close-packed hexagonal arrays. As the solvent evaporates the distance between the MG peripheries increases as the MG particles deswell.^22^ The particles remain separated from each other within non-close-packed hexagonal arrays at the air/liquid interface as solvent evaporation continues. Finally, the arrays of MGs are transferred to the underlying solid substrate as a consequence of complete evaporation of the solvent.^22^

Our group has used PNP MGs as sacrificial templates to introduce controlled porosity into meso-TiO_2_ layers of PSCs.^42^ Those sacrificial MGs were burnt out of the meso-TiO_2_ layer using heating at 400 °C. More recently, we showed in a proof-of-concept study that ordered non-close-packed 2D arrays of PNP MGs could be formed within the perovskite layer during precursor deposition.^43^ The perovskite used in that study was a MAFA-based perovskite (FA_0.75_MA_0.25_)PbI_3_. Those STPSCs were red-brown in color. Whilst limited structural order was present, the distance between MGs was either too large or the extent of order was too low to give structural color. Furthermore, the stability of the devices or films was not investigated. Here, we show that new PNP MGs overcome those problems and provide highly structured arrays within two different perovskites which we use to demonstrate structurally colored STPSCs. We also show that inclusion of the MGs strongly improves the stability of the devices and provide a conceptual theory to explain this result.

**Scheme 1:**
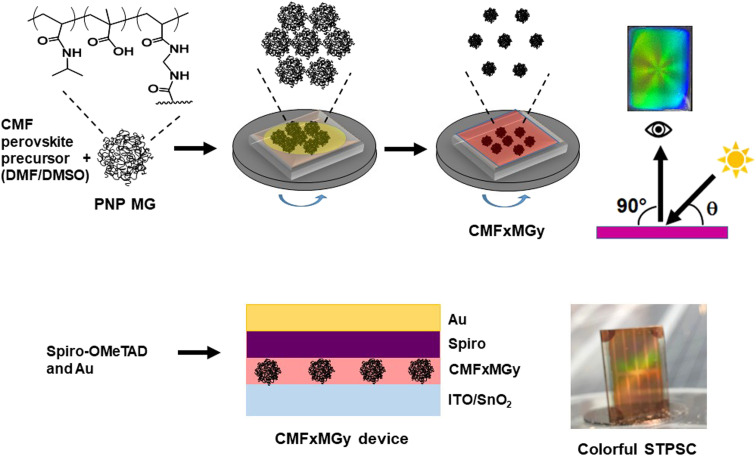
Depiction of the procedure followed for making colorful semitransparent perovskite films and devices. The films and n–i–p STPSCs are colored in reflection. CMF*x*MG*y* represents the MG-based triple-cation perovskite. The values of *x* and *y* are the concentrations of perovskite and MG used in the precursor solution.

In this study, we exploit the self-ordering of PNP MGs to produce structural color within STPSCs (Scheme 1). We show that the reflected color is tuneable *via* control of the formulation used to deposit the perovskite layer. A simple ray tracing theory is developed that enables prediction of the color of the films and devices. We achieve a very good PCE value (10.60%) for a structurally colored STPSC with an AVT greater than 25%. We establish the generality of our approach using two different perovskites (double-cation and triple-cation) that have not previously been used with PNP MGs. The results also show that the stability of the STPSC devices is enhanced by the PNP MGs. We demonstrate that the structural color can be used to visually assess the stability of the films. This study introduces a new scalable one-step method for preparing structurally colored STPSCs that may bring forward deployment of perovskite-based systems for BIPV applications.

## Results and discussion

### Characterization of the PNP MG particles

**Fig. 1:**
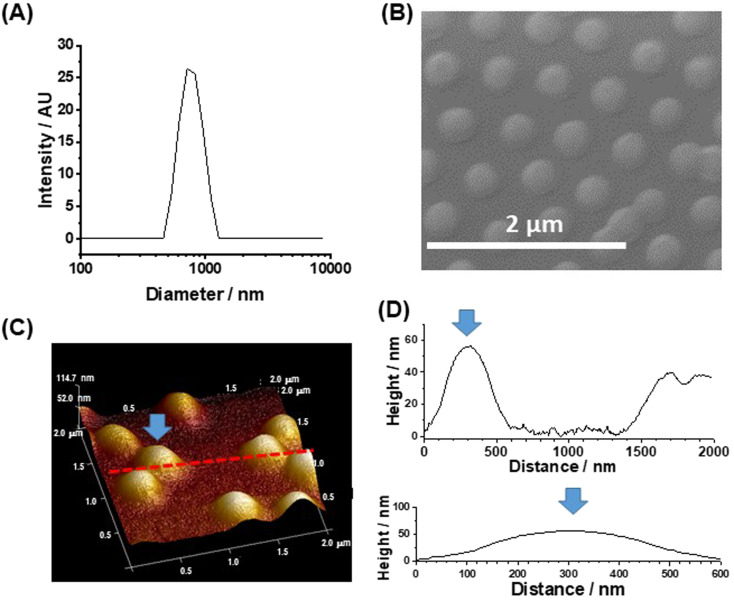
(A) Dynamic light scattering (DLS) diameter distribution for the MGs in DMF/DMSO. (B) SEM image of MGs deposited from DMF/DMSO (4 : 1) on glass/ITO/SnO_2_. (C) Perspective AFM image of the particles. The arrow and line show the features in the line profile analysis in (D) the latter also shows a plot whereby the *x*- and *y*-axis values have the same linear distances.

Three changes were made to the preparation of the MGs in this study compared to those used in our earlier work.^43^ The PNP MGs used here were prepared using a higher methacrylic acid content, a higher total particle concentration and a lower crosslinker content. The MGs had a *z*-average diameter (*d*_*z*_) of 763 nm when dispersed in DMF/DMSO (Fig. 1A). A representative SEM of deposited MGs gave a number-average diameter of 352 ± 41 nm (Fig. 1B). Comparison of the SEM and DLS data shows that the MGs swell in mixed DMF/DMSO solvent which is a criterion for successful MG use in perovskite films. Tapping-mode AFM data (Fig. 1C and D) show that the deposited particles had a diameter of ∼600 nm and a height of 56 nm. The AFM diameter is larger than measured from SEM because the periphery of the particle is very thin (<20 nm) and has low electron contrast when viewed by SEM, which is shown by the line profiles (Fig. 1D). The high aspect ratio (diameter-to-height ratio) for our MGs of ∼10 reveals that they flattened considerably upon deposition, which has been reported previously for related PNP MGs^44^ on other substrates.

### Morphology of the structured perovskite-MG films

**Fig. 2:**
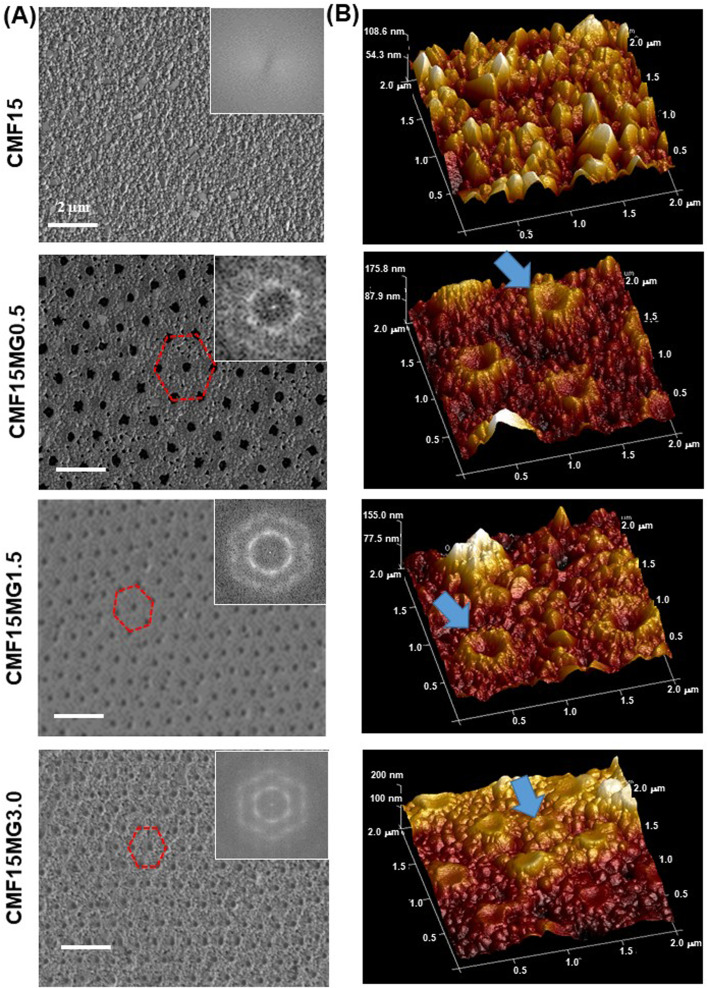
(A) Top view SEM images with FFT images and (B) AFM perspective height images of various films. Hexagonal non-close-packed pores are highlighted in (A). MGs are highlighted in (B).

A preliminary study showed that the use of the triple cation-based perovskite (Cs_0.05_MA_0.02_FA_0.93_)Pb(I_0.98_Br_0.02_)_3_ films prepared with 15 wt% perovskite and various PNP MG concentrations (CMF15MG*y*) gave composite films with AVTs well above 25% (Fig. S1, ESI†). The use of these films resulted in devices with AVTs that were above 25% (see below). Inclusion of the MGs into the precursor solutions provides perovskite films with non-close-packed hexagonal pores with high extents of structural order (Fig. 2A). The respective FFT images (insets) show well defined hexagonal symmetry. Higher magnification SEM images showed that the average perovskite grain sizes for the CMF15MG*y* films (where, *y* = 0, 0.5, 1.5 and 3.0) were 148 ± 60, 132 ± 36, 129 ± 41 and 119 ± 39 nm, respectively (Fig. S2, ESI†). The period (*D*) is the average distance between the centres of nearest neighbor particles (Fig. S3A, ESI†). To probe the relationship between *D* and *y*, a CMF15MG1.0 film was also prepared (Fig. S3B, ESI†). The value for *D* decreases as the MG concentration increases (Fig. S3C, ESI†). An empirical fit of the period *vs.* MG concentration data (Fig. S3D, ESI†) shows that the following equation applies for the CMF15MG*y* films:

1 *D* = 1008*y*^−0.39^

where *y* is the MG concentration used. This empirical equation is important because it establishes a link between the period and formulation conditions used to deposit the films.

AFM 3D perspective images (Fig. 2B) show well-resolved concave depressions (pores) where the MGs reside. The concave shape of the pores implies that the MGs wetted the perovskite as the particles deswelled during solvent evaporation (see Fig. S4, ESI†). Using a cake baking analogy, the situation resembles a Victoria sponge cake that deflates in the middle but still grips the baking pan at the circumference (see cartoon in Fig. S4, ESI†). SEM images of cross-sections of films (Fig. S5, ESI†) show the pores have a dual concave morphology as revealed by the expanded view for the CMF15MG1.5 system. The film thickness values measured from the SEM images for the CMF15MG*y* films (where, *y* = 0, 0.5, 1.5 and 3.0) are 90, 117, 127 and 134 nm, respectively.

The mechanism for formation of the pores begins with transfer of the MGs (discussed in the Introduction) from the precursor air/solution interface to the ETL. Perovskite crystallization then surrounds the deposited MGs. The subsequent evaporation of solvent from the laterally constrained MGs causes their vertical collapse and formation of pores within the perovskite. The concave structure of the MGs within the pores (Fig. S4 and S5, ESI†) and their contact with the perovskite at the pore extremities implies that an attractive MG-to-perovskite interaction occurred.

An alternative explanation for the morphologies in Fig. 2 is that the antisolvent partially dissolved or washed away the MGs. We note that the MGs are crosslinked particles that swell, but do not dissolve, in good solvents such as DMF. However, CBZ (the antisolvent) is a poor solvent for PNP. Consequently, the antisolvent treatment should not have partially dissolved or swelled these MGs. Indeed, a SEM study (Fig. S13, ESI†) of degraded films (discussed below) shows that the MGs remained on the ETL after the perovskite degraded, which confirms the MGs were not washed away.

### Investigating structural color of semitransparent perovskite-MG films

**Fig. 3:**
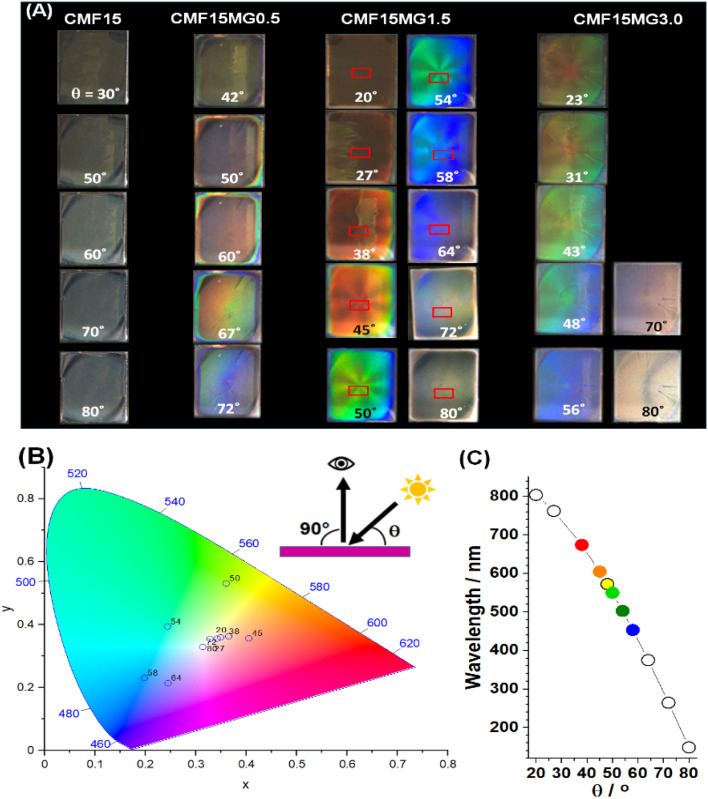
(A) Photographs taken of reflected color for CMF15MG*y* films at variable incident angles (*θ*). The geometry of the experiment is depicted. (B) *xy* color coordinates obtained from the sampled areas shown in rectangles from the CMF15MG1.5 films in (A). (C) Wavelengths of the reflected light (*λ*) for the CMF15MG1.5 film calculated using eqn (2) at the *θ* values used in (A).

We investigated the effect of incident light angle (*θ*) on the reflected light color (Fig. 3A and B). All the films containing MGs showed reflected color at *θ* values between 20 and 80°. (Details of the positions of the light source and observer are provided in Fig. S6, ESI†).

All colors of the spectrum can be seen from examination of the films shown in Fig. 3A. The color pallet available is not restricted for these new structurally colored perovskite films. The *θ* region where blue reflected color was observed moved to lower *θ* values as the MG concentration increased (and *D* decreased), *i.e.*, from 72° for CMF15MG0.5, to 58°–64° for CMF15MG1.5 to 56° for CMF15MG3.0. Regions of the CMF15MG1.5 images were sampled (red rectangles in Fig. 3A) and converted to CIE 1931 *xy* coordinates using a bespoke image analysis program and the data plotted (Fig. 3B). This figure confirms that a wide variety of reflected colors is accessible.

We applied ray tracing to the geometry studied (see Additional Note 1 and Fig. S7, ESI†) and derived the following predictive equation for the reflected wavelength (*λ*) and hence the color apparent for these new structurally colored perovskite films.

2 *λ* = *D* cos *θ*

Values for *λ* are estimated using eqn (2) for CMF15MG1.5 and the values are shown in Fig. 3C. The *λ* values generally agree with the colors observed in Fig. 3A. Hence, eqn (2) captures the physics of the colored films shown in Fig. 3A because: (1) for fixed *D*, *λ* increases (moving from blue to red) as *θ* decreases. (2) As *D* decreases, *λ* decreases (moving to blue) at fixed *θ*. (3) As *D* decreases (*e.g.*, as *y* increases for CMF15MG*y*), the value for *θ* moves to smaller values at fixed *λ* (*i.e.*, at constant color). Crucially, because *D* is related to MG concentration according to eqn (1), the combination of eqn (1) and (2) provides color tuneability for a desired *λ* at a specific angle *via* the CMF15MG*y* precursor MG concentration according to:

3 *y* = (1008 cos *θ*/*λ*)^2.56^

### Structurally colored semitransparent perovskite-MG solar cells

**Fig. 4:**
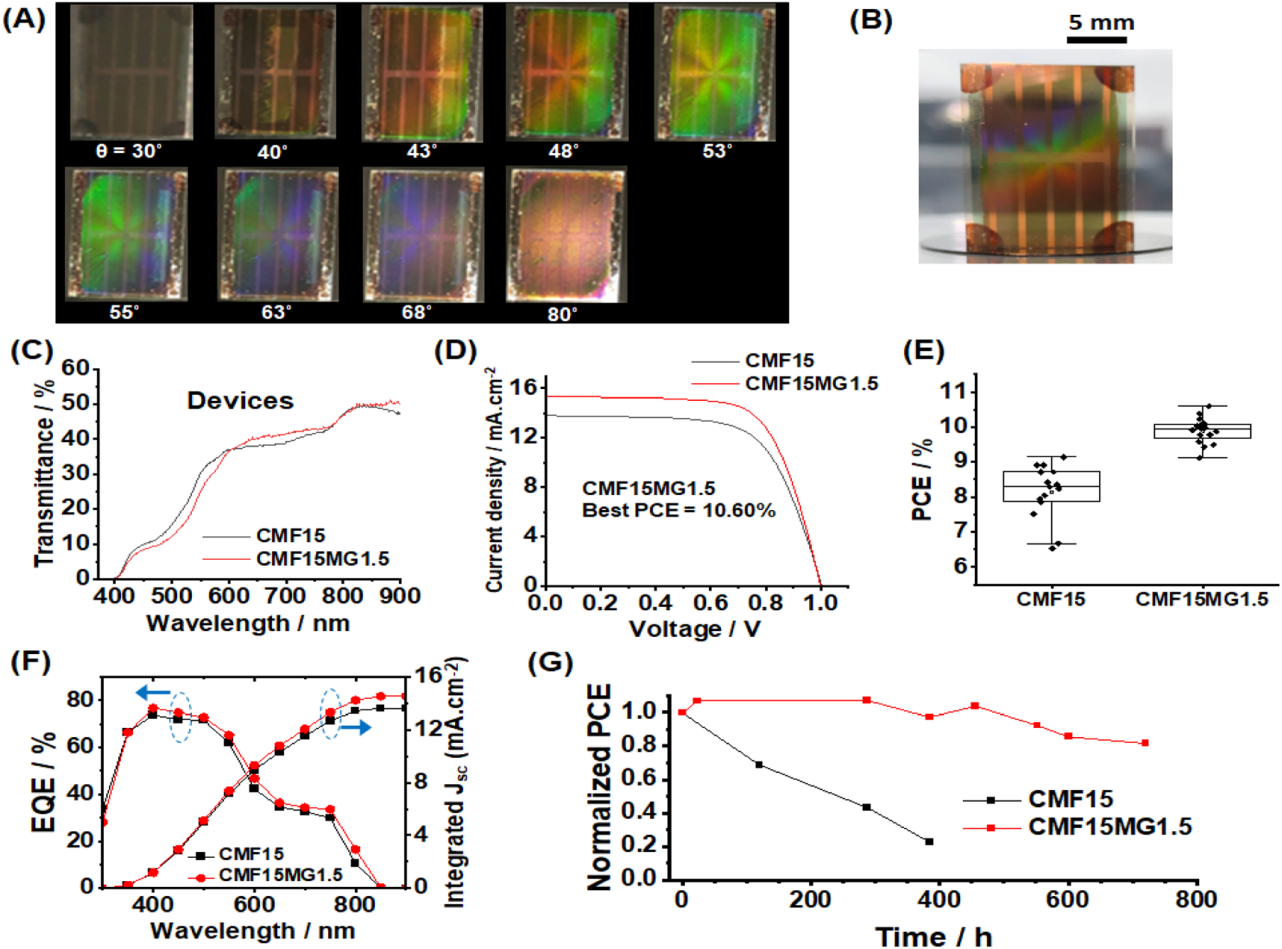
(A) Photographs of CMF15MG1.5 devices obtained from reflected light. The *θ* values are shown. (B) Photograph of a CMF15MG1.5 solar cell demonstrating semitransparency and color. (C) Transmittance spectra of the devices. (D) *J*–*V* curves for the devices. (E) Box plots of the PCE data for the devices. (F) EQE spectra. (G) PCE stability for the control CMF15 and CMF15MG1.5 devices. The unencapsulated devices were stored in the dark at ∼50% RH at room temperature.

To demonstrate the potential application as a colored STPSC, we constructed devices using CMF15MG1.5 because these films gave the strongest reflected colors visually as well as a relatively broad angle range over which color can be seen (Fig. 3A). (The device architecture is shown in Scheme 1.) The CMF15MG1.5 device was colorful in reflected light over the *θ* range of 43–68° (Fig. 4A). Fig. 4B shows that the new device was both colorful and semitransparent. A CIE diagram was constructed using image analysis of the device images (Fig. S8A and B, ESI†). Furthermore, the values for *λ* calculated by eqn (2) generally agree with those observed visually (Fig. S8C, ESI†). The CIE diagram (Fig. S8B, ESI†) shows that the color depth decreased compared to the parent films (Fig. 3B), which is due to the spiro-OMeTAD and Au layers. Indeed, transmittance spectra for the Au and spiro-OMeTAD films (Fig. S9, ESI†) show that the former decreases transmission of light at 600–740 nm^45^ (orange to red region) whilst the latter decreases transmission below 425 nm (violet region). In principle, transparent top contacts and HTM optimization could be used to increase the color depth of these new structurally colored STPSCs. The transmittance spectra measured for the CMF15MG1.5 and CMF15 devices (Fig. 4C) gave AVTs of 25.5% and 25.4%, respectively, which are above 25%.

*J*–*V* data are shown for the CMF15MG1.5 and CMF15 devices in Fig. 4D and box plots for the PCE appear in Fig. 4E. (All the data are summarized in Table S1, ESI†). The champion CMF15MG1.5 and CMF15 STPSCs had PCE values of 10.60% and 9.14%, respectively. Box plots of the other performance parameters (Fig. S10, ESI†) show that the improved PCE of CMF15MG1.5 compared to CMF15 is due to increased *J*_sc_ and FF. The increased *J*_sc_ may be due improved light management afforded by the MGs as reported earlier for related (non-colorful) STPSCs.^43^ EQE spectra (Fig. 4F) show that these devices harvest the most photons from the violet-to-green parts of the spectrum. This is more pronounced for the CMF15MG1.5 device. The integrated *J*_sc_ values from the EQE spectra agreed with those obtained from the *J*–*V* curves to within 5%. The improved FF of the CMF15MG1.5 devices compared to CMF15 is attributed to less recombination.

Steady-state PL spectroscopy was used to probe the films (Fig. S11A and B, ESI†). The PL intensity increased as the MG content increased. This trend is indicative of passivation of the perovskite^46^ by the MGs. Time-resolved PL (TRPL) measurements were also made (Fig. S11C, D and Table S2, ESI†). The increase in the average charge carrier lifetime confirms that passivation of defects was provided by the MGs.

We studied the stability of the devices using dark storage tests (Fig. 4G). The data show that the CMF15MG1.5 device retained 82% of the initial PCE after 720 h (30 days). In contrast, the control device lost more than 77% of the initial PCE after 384 h (16 days). The effect of a humid atmosphere on the stability of the films was also investigated (Fig. S12, ESI†). The photographs obtained with incident light normal to the films (non-colored viewing mode) show that the CMF15MG1.5 film retained its brown color after 24 h; whereas the control was colorless. Hence, CMF15MG1.5 was more stable to humid air than CMF15. The reflected light photographs (color viewing mode, Fig. S12, ESI†) show that the color intensity fades with time and almost completely vanishes after 210 h exposure to 90% RH air. A representative SEM image (Fig. S13, ESI†) reveals that the perovskite degraded substantially after 210 h (compare with Fig. 2A). Accordingly, the strong fading of the color during degradation (Fig. S12, ESI†) is due to the decrease in refractive index difference between the MGs and the matrix as the perovskite surrounding the self-ordered MGs is replaced by air.

### Origin of the stability improvement provided by the MGs

PNP MGs dispersed in water are known to expel water when the temperature exceeds the lower critical solution temperature (LCST). At such temperatures, hydrophobic isopropyl groups dominate interactions.^47^ The LCST can be decreased from 32 °C for pure water to lower temperatures by adding salt to the solution.^48^ We measured the *d*_*z*_ values for the MG dispersed in water containing a range of FAI concentrations (Fig. S14, ESI†). The value for *d*_*z*_ decreased strongly which is due to a decrease of the LCST. These data show that FAI decreases the LCST of the MGs triggering a hydrophilic-to-hydrophobic transition. We propose that this transition contributes to the stability protection when water comes into contact with the MGs embedded within STPSCs. Hence, the MGs provide a “smart” defence system that becomes activated when water is present.

### Generality of imparting color to STPSCs using PNP MGs

**Fig. 5:**
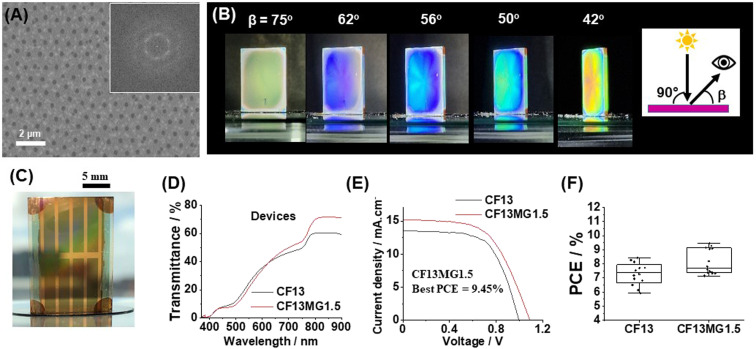
(A) Top view SEM image for a CF13MG1.5 film. The inset shows the FFT image. (B) Iridescence photographs for a CF13MG1.5 film viewed at different angles of reflected light (*β*). The incident light was normal to the film (*θ* = 90°). (C) A photograph of a colorful semitransparent CF13MG1.5 device. (D) Transmittance spectra of the CF13 and CF13MG1.5 devices. (E) *J*–*V* curves of the CF13 and CF13MG1.5 devices. (F) Box plots for the PCE data obtained.

We tested the generality of our new approach by using a double cation perovskite with the composition (Cs_0.12_FA_0.88_)Pb(I_0.92_Br_0.08_)_3_. The latter is abbreviated as CF. A preliminary study showed that CF13MG*y* systems provided AVTs sufficiently high that STPSCs would have AVTs above 25% (Fig. S15, ESI†). Non-close-packed MGs are apparent from the SEM image of CF13MG1.5 with a high degree of order (Fig. 5A). The films are iridescent as shown by photographs taken when the viewing angle (*β*) is changed at constant angle of incident light (*θ* = 90°, Fig. 5B). The CF13MG1.5 devices also gave color when used as a window segment (Fig. 5C) and as a function of *θ* (Fig. S16, ESI†). The transmission spectra (Fig. 5D) gave AVT values for the CF13MG1.5 and CF13 devices of 27.1% and 27.0%, respectively, which are both above 25%. The *J*–*V* data (Fig. 5E) gave PCEs for champion CF13MG1.5 and CF13 STPSCs of 9.45% and 8.44%, respectively. Box plots for the PCE are shown in Fig. 5F and the other parameters in Fig. S17 (ESI).† (The device performance parameters are also shown in Table S1, ESI†). The integrated *J*_sc_ values from the EQE spectra (Fig. S18, ESI†) agree with those from the *J*–*V* measurements. The MG containing system has a higher PCE than the control. This is primarily due to the higher *V*_oc_ for the MG system (Table S1, ESI†). PL data for the CF13MG1.5 and CF13 films (Fig. S19, ESI†) show larger PL intensity and decay times for the CF13MG1.5 system compared to the CF13 control which favors a higher *V*_oc_. Hence, our approach for preparing colorful STPSCs can be applied to more than one perovskite and is generally applicable. Future work will involve scaling up our approach to larger area devices.

## Conclusions

In this study, we have demonstrated a new scalable one-step method for preparing structurally colored STPSCs. This work has shown that spontaneous self-ordering of MGs within the perovskite lattice can give structurally colored STPSCs and that the color can be tuned by control of the period according to eqn (2). Furthermore, eqn (3) is a predictive equation for the value of *y* to use for the CMF15MG*y* systems to reflect a particular wavelength (and hence color) at a specific incident angle. This study has shown that structural color and iridescence can be achieved from perovskite films containing PNP MGs provided the structural order of the non-close packed hexagonal arrays is high and *D* is in the range of 670–1350 nm. The color gamut translates successfully to the devices. This study represents the first example of self-assembly of a colloidal additive during one-step perovskite film deposition as a method to provide structurally colored STPSCs. Our method does not require any additional processing steps. The generality of our new approach was demonstrated using two different perovskites. The best PCE achieved was 10.60% for the CMF15MG1.5 which had an AVT of 25.5%. The latter was much higher than that for CMF15 control (best PCE = 9.14% and AVT = 25.4%). Not only did the MGs provide structural color, but they increased the PCE and device stability. The simplicity, generality and scalability of our new approach may bring forward deployment of STPSCs.

## Author contributions

OMA conducted most of the experiments and made the devices and helped with preparation of the manuscript. RW helped with the preparation of perovskite precursor solutions. ZJ performed the gold coating and PCE measurements for the devices. NWH conducted the AFM measurements. A. Alruwaili performed the XRD measurements and helped with analysis. A. Altujjar helped with the SEM and TRPL measurements. EP developed the method and code required and provided the color coordinates for CIE chromaticity diagrams. BRS wrote most of the manuscript and directed the research.

## Conflicts of interest

There are no conflicts to declare.

## Supplementary Material

RA-014-D4RA00324A-s001
