# Supplementary material for: Structurally colored semitransparent perovskite solar cells using one-step deposition of self-ordering microgel particles
Source: RSC Adv. 2024 Feb 19;14(9):6190–8. doi: 10.1039/d4ra00324a (PMC10875278; doi:10.1039/d4ra00324a)
Supplement: RA-014-D4RA00324A-s001 [file RA-014-D4RA00324A-s001.pdf]

Electronic Supplementary Material (ESI) for RSC Advances.
This journal is © The Royal Society of Chemistry 2024

SUPPORTING INFORMATION

Structurally colored semitransparent perovskite solar cells using one-step deposition of self-ordering microgel particles

Osama M. Alkhudhari[a,]*, Ran Wang[a], Zhenyu Jia[a], Nigel W. Hodson[b], Amal Alruwaili[a], Amal Altujjar[a,c], Eugenio Picheo[a] and Brian R. Saunders[a,]*

a) *Department of Materials, University of Manchester, Engineering Building A, Manchester, M1 7HL, U.K.*

b) *BioAFM Facility, Faculty of Biology, Medicine and Health, Stopford Building, University of Manchester, Oxford Road, Manchester, M13 9PT, U.K.*

c) *Basic Science Department, Deanship of Preparatory Year and Supporting Studies, Imam Abdulrahman Bin Faisal University, Dammam 34221, KSA.*

EXPERIMENTAL DETAILS

Materials

Methacrylic acid (MAA, 98%), *N*-isopropylacrylamide (NP) (≥ 99%), methylenebisacrylamide (BIS, 99%), potassium persulfate (KPS, 99%), and sodium dodecyl sulphate (SDS, 98.5%) were all purchased from Sigma-Aldrich and used as received. Methylammonium chloride (MACl, 99%), $PbBr_2$ (99.99%), CsI (99.9%), titanium isopropoxide (TIP, 97%), 2-methoxy ethanol (99.8%), ethanolamine (≥ 99.5%,), 2,2',7,7'-tetrakis[*N*,*N*-di(4-methoxyphenyl)amino]-9.9'-spirobifluorene (Spiro-OMeTAD, 99%), 4-tert-butylpyridine (TBP, 96%), and bis(trifluoromethane) sulfonimide lithium salt (LiTFSI, 99.95%) were also all purchased from Sigma-Aldrich. Methylammonium bromide (MABr, 98%), formamidinium iodide (FAI, 98%, Ossila), and PbI_2 (99.99%, TCI) were used as received. *N*,*N*-dimethylformamide (DMF, 99.8%), dimethyl sulfoxide (DMSO, 99%), tris(2-(1H-pyrazol-1-yl)-4-tert-butylpyridine) cobalt(III) tri[bis(trifluoromethane) sulfonimide] (FK 209 Co(III) TFSI), acetonitrile (99.9%), chlorobenzene (CBZ, 99.8%), and 2-propanol (IPA, 99.5%) were all purchased from Acros. Acetone (ACS reagent, ≥ 99.5%), isopropyl alcohol (IPA, ≥ 99.5%) and ethanol (EtOH, ≥99.5%) were purchased from Fisher Scientific and used as received. All water used was ultrahigh purity and deionized.

Microgel synthesis

The synthesis of PNP MG was conducted using a precipitation polymerization method reported elsewhere[1]. NP (0.40 g, 3.53 mmol), SDS (5.4 mg), BIS (2.0 mg, 0.013 mmol), and MAA (0.052 g, 0.60 mmol) were dissolved in 20 mL of water in a 250 mL three-neck round-bottom flask. The reactor was degassed by N_2 purge for 1 h followed by addition of 0.012 g of KPS solution dissolved in 5.0 mL of water. Then, the glass reactor was kept under N_2 atmosphere with vigorous stirring at 70 °C for 7 h. The mixture was left to cool down to room temperature. For microgel purification, two repeated centrifugation and redispersion cycles in water were carried out. The MG particles were then transferred from water into mixed solvents (DMF/DMSO) by repeated centrifugation and

redispersion and finally diluted to a concentration of 5.0 wt. % in DMF/DMSO (4:1).

Preparation of triple cation-based perovskite precursor solution

The chemical formula used for the triple-cation perovskite is $(Cs_{0.05}MA_{0.02}FA_{0.93})Pb(I_{0.98}Br_{0.02})_3$. This perovskite is abbreviated as CMF. The precursor solution (1.5 M) was prepared by mixing MACl (0.0105 g, 0.16 mmol), CsI (0.013 g, 0.05 mmol), FAI (0.16 g, 0.93 mmol), MABr (0.00213 g, 0.02 mmol), $PbBr_2$ (0.0075 g, 0.02 mmol), and PbI_2 (0.475 g, 1.03 mmol) in a mixed solvent of DMF/DMSO (4:1) (0.667 mL) followed by heating at 50 ºC for 2 h with vigorous stirring. The precursor was then filtered using a 0.2 µm syringe filter. CMFxMGy represent the MG-based triple-cation perovskite films. The values of *x* and *y* are the concentrations of perovskite and MG used in the precursor solution, respectively. The concentration of the stock CMF and MG solutions were 51.0 wt% and 5.0 wt%, respectively. The following gives an example of the preparation of the precursor solution for a CMF15MG1.5 film. To a CMF solution (29.4 µL), MG solution (30 µL) and DMF/DMSO mixed solvent (4:1) (40.6 µL) were added and mixed thoroughly.

Preparation of the double cation-based perovskite precursor solution

The chemical formula used for the double-cation mixed-halide perovskite that was also used in this study is $(Cs_{0.12}FA_{0.88})Pb(I_{0.92}Br_{0.08})_3$. A 1.3 M precursor solution of CsI_2 (0.033g, 0.13 mmol), MACl (0.022 g, 0.33 mmol), FAI (0.16 g, 0.93 mmol), $PbBr_2$ (0.050 g, 0.14 mmol) and PbI_2 (0.50 g, 1.085 mmol) were dissolved in a mixed solvent of DMF/DMSO (4:1) (0.79 mL). The precursor solution was then heated on pre-hot plate at 50 ºC for 90 min with vigorous stirring. Then, a syringe filter (0.2 µm) was used to filter the mixture before the spin-coating process. CFxMGy is used to denote MG-double cation composite films containing microgel, where CF represents the perovskite, MG is the PNP microgel, *x*, and *y* are the concentration in wt.% of the CF perovskite precursor and the MG, respectively. The concentration of the stock CMF and MG solutions were 50.0 wt% and 5.0 wt%, respectively. The following gives an example of the preparation of a precursor solution for a

CF13MG1.5 film. To a CF solution (26.0 μL), MG solution (30 μL) and DMF/DMSO (4:1) (44.0 μL) were added and mixed thoroughly.

Triple cation-based perovskite film fabrication

The substrate used for the triple-cation was unpatterned ITO. Ultrasonication in water (containing 1.0 wt.% Hellmanex solution), acetone and IPA (5 minutes each) were used, sequentially, to wash the ITO substates. N_2 was used to dry substrates followed by a UV-ozone treatment for 15 min. SnO_2 (3.25%) was employed as the ETL. It was prepared by diluting 356 μL of SnO_2 (15.0%) in 1644 μL of water followed by heating at 35 ºC for 20 min and filtration by a 0.2 μm syringe filter. The SnO_2 layer was then deposited on the ITO substrate at 3000 rpm for 30 s. A pre-heated hot plate was used to anneal the ITO/SnO_2 substrates that were heated at 150 °C for 30 min and then treated with UV-ozone for 10 min. CMFxMGy precursor solution (85 μL) was coated onto SnO_2 by applying (1) 1000 rpm for 10 s and (2) 6000 rpm for 20 s with the addition of an antisolvent (CBZ, 200 μL) in the last 10 s of the process. On a pre-heated hotplate at 120 ºC the ITO/SnO_2/CMFxMGy film was placed and annealed for 40 min to form the colorful film.

Double cation-based perovskite film fabrication

For the double cation-based perovskite film a blocking layer (bl) TiO_2 film was deposited onto a FTO-coated glass substrate by mixing 160 μL of TIP and 140 μL of ethanolamine in 2.0 mL of 2- methoxy ethanol. After heating the mixture at 45 °C for 5 min, it was filtered using a syringe filter (0.2 μm). The solution (100 μL) was spin coated onto the FTO substrate at 5000 rpm for 30 s with an initial acceleration of 2000 rpm/s. A pre-heated hotplate at 450 °C was used to heat the FTO/bl-TiO_2 substrate for 45 min and the substrate subsequently cooled to room temperature. CFxMGy precursor solution (85 μL) was coated on the bl-TiO_2 by applying 1000 rpm for 10 s and 4000 rpm for 30 s followed by the addition of CBZ (200 μL) antisolvent at the end of 10 s of the second step. On a hotplate pre-heated to 100 °C the FTO/bl-TiO_2/CFxMGy was annealed for 50 min to form the colorful film.

Solar cell construction

The procedure used to prepare the devices after the perovskite films had been formed is common for both perovskite types. The HTL was prepared by diluting 0.090 g of Spiro-OMeTAD, 34 μL of TBP, 19 μL of LiTFSI solution in acetonitrile (0.52 $g.mL^{-1}$) and 10 μL of FK209 solution in acetonitrile (0.30 $g.mL^{-1}$) in 1.0 mL of CBZ. A 0.2 μm syringe filter was used to filter the Spiro-OMeTAD solution after it was heated to 45 °C for 5 min. Then, the HTL precursor solution (80 μL) was dynamically spin-coated onto the perovskite layer at 3000 rpm for 30 s. For 24 h, the samples were kept in a dry, dark environment to allow oxidation of the Spiro-OMeTAD. Ultimately, using thermal evaporation, an ultra-thin Au layer (10 nm) was coated on top of the Spiro layer to form the final structure of the *n-i-p* colorful STPSCs.

Physical measurements

A Malvern Nano ZS instrument was utilized for dynamic light scattering (DLS) to measure the z-average diameter (d_z). UV-visible spectra were measured using a Perkin Elmer lambda 25 spectrometer. The AVT values were calculated by measuring the average transmittance in the wavelength range of 380 to 760 nm[2, 3]. An Edinburgh instrument FLS980 was employed to measure photoluminescence (PL) spectra and time-resolved photoluminescence (TRPL) data with the incident light on the glass side. The excitation wavelength was 470 nm. A long-pass filter at 590 nm was used. An excitation wavelength of 405 nm and a pulse period at 200 nm was used for the TRPL measurements. For top-view and cross-sections SEM imaging, a Zeiss Ultra 55 was utilized with an electron high tension (EHT) of 1.5 kV. A secondary electron (SE) detector was applied for all the top-view SEM images while an energy selective backscattered (ESB) electron detector was used for the cross-sections SEM images. AFM images were obtained using a Bruker Bioscope Catalyst AFM with a Nanoscope V controller (Bruker UK Ltd., Coventry, UK), operating under the Bruker Nanoscope controller software (v9.15), and mounted on a Nikon Eclipse Ti-I optical microscope (Nikon Instruments Europe B.V.). A ScanAsystTM (peak-force tapping) mode with ScanAsyst Air probes were used to capture Images in air. A Bruker Nanoscope Analysis (v3) software package

(Bruker Corporation, USA) was utilized for Image analysis. XRD diffraction data were achieved using a Malvern PANalytical XRD 5. The XRD data were analyzed using Xpert HighScore software. Two mobile phones, (an iPhone 8 and Samsung S22), were used to capture photographs of the colorful films. The Samsung S22 mobile phone was used as the light source and the iPhone 8 was used as the camera as depicted in Fig. S6. For moisture stability measurements, a moisture chamber at ~90% RH was utilized.

Color characterization coding

To plot the 1931 CIE colour diagrams a Python code was used to convert the colour distribution of selected regions of input digital images of the films to *xy* colour space coordinates, using a Anaconda3 Jupyter Notebook. For each digital photograph the jpg image file was converted to a pixel array using Image python package[4]. The red-blue-green (RGB) values of the selected pixels of interest were converted to tristimulus XYZ values. This involved X (CIE RGB curves), Y (luminance), Z (quasi-equal blue) conversion to *xy* coordinates of 1931 CIE color diagram using RGB to XYZ and XYZ to *xy* conversion matrices respectively, from Color open-source Python package[5]. The *xy* coordinates, stored in text file, were plotted in CIE diagrams using Origin.

Solar cell characterization

Using an ABET solar simulator, the current-density voltage characteristics were measured via a Keithley 2420 Sourcemeter with AM 1.5G illumination (100 $mW.cm^{-2}$). The instrument was calibrated and corrected for spectral irradiance mismatch using a certified Oriel Si-reference cell. A square aperture was used to define the device area, which is 0.079 cm^2. The illumination was from the glass side for all devices. The PCEs and other data studied in this work are obtained from the reverse scans unless otherwise stated. A Newport QuantX-300 instrument was used for the EQE measurements.

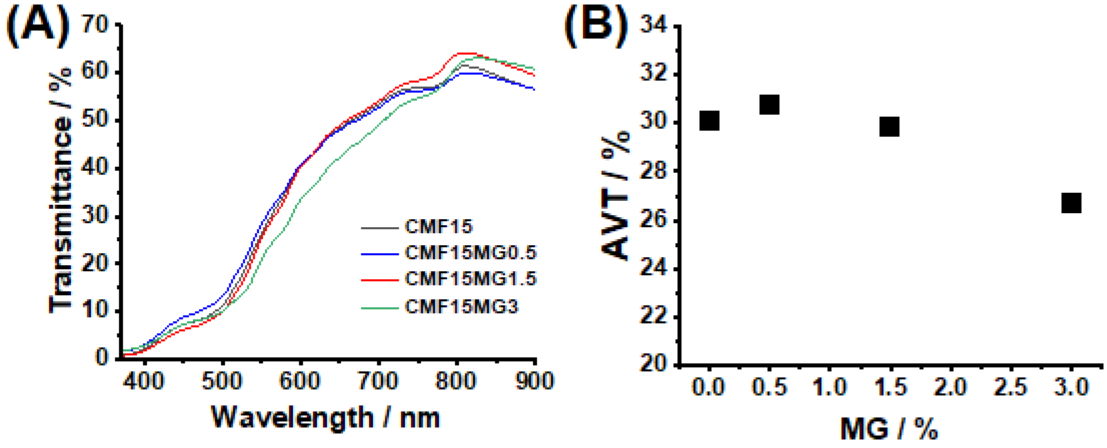

Fig. S1. (A) Transmittance spectra for various CMF15MGy films and **(B)** average visible transmittance data obtained from the spectra in (A).

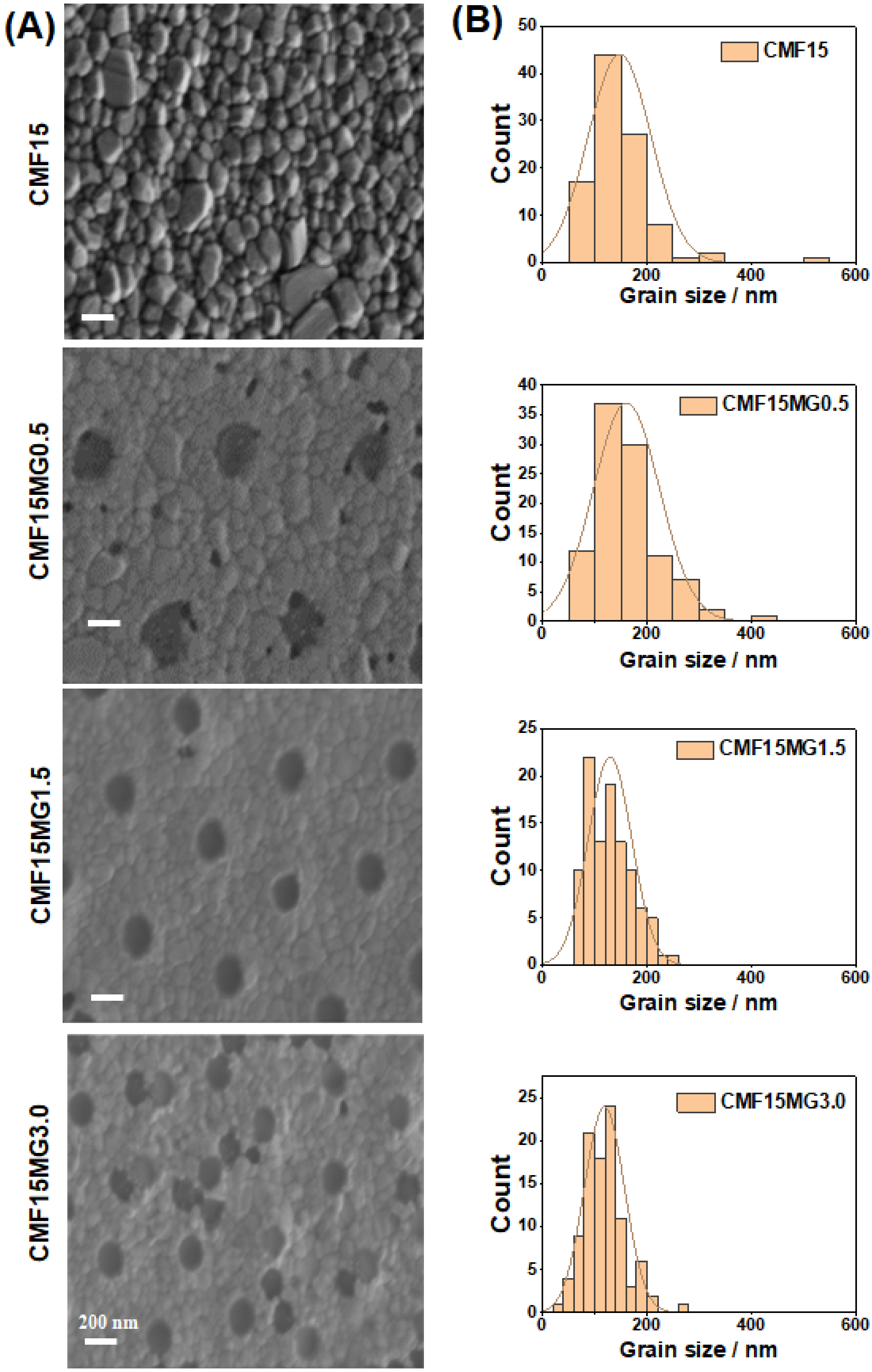

Fig. S2. (A) Higher magnification SEM images and **(B)** grain size distributions for various films.

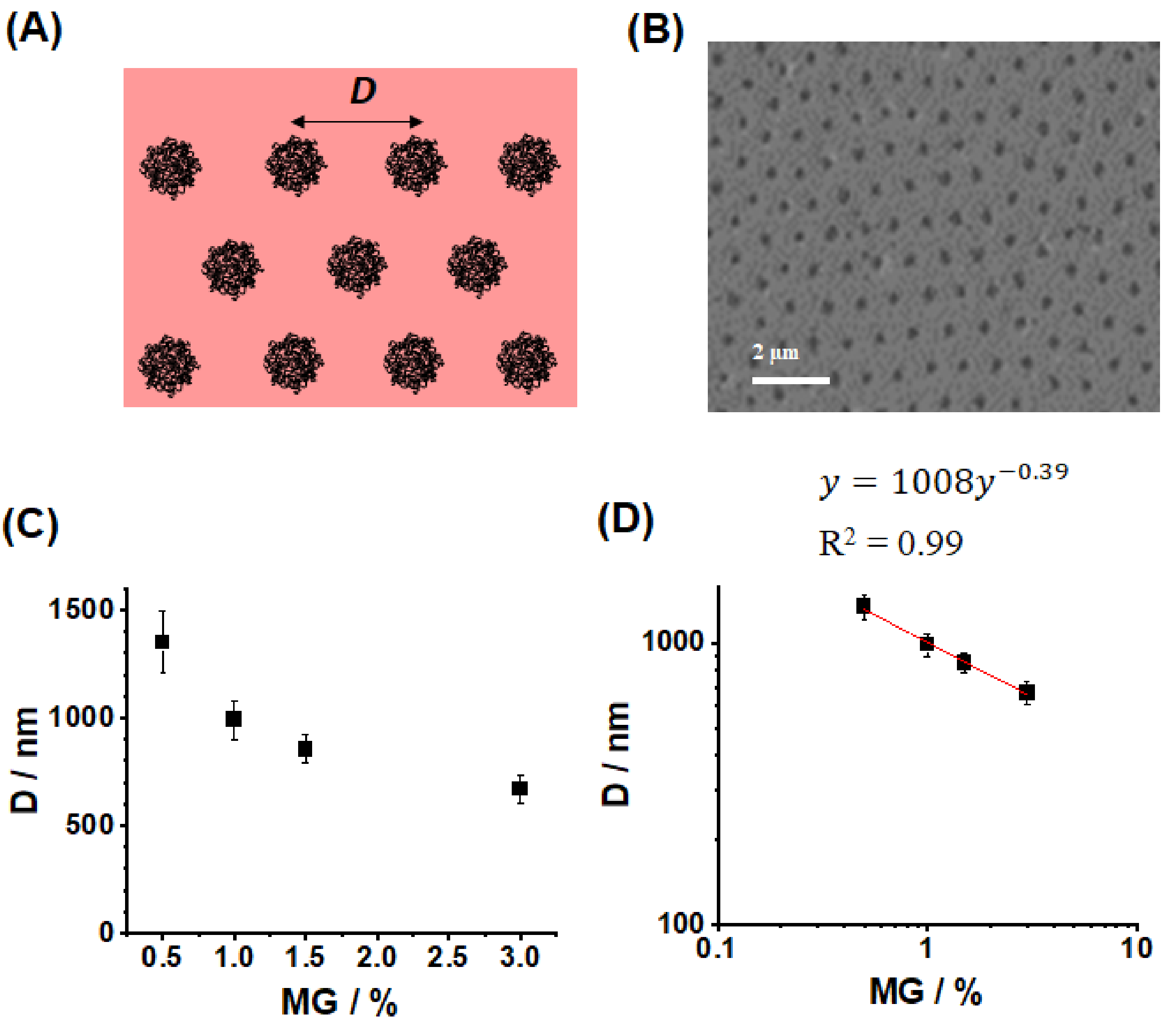

Fig. S3. (A) A cartoon depicting the period as the separation between MG centers. **(B)** SEM image for a CMF15MG1.0 film. **(C)** Variation of the period (*D*) for the array of MGs in the CMF15MGy films with MG concentration. **(D)** A log-log plot of the data from (C).

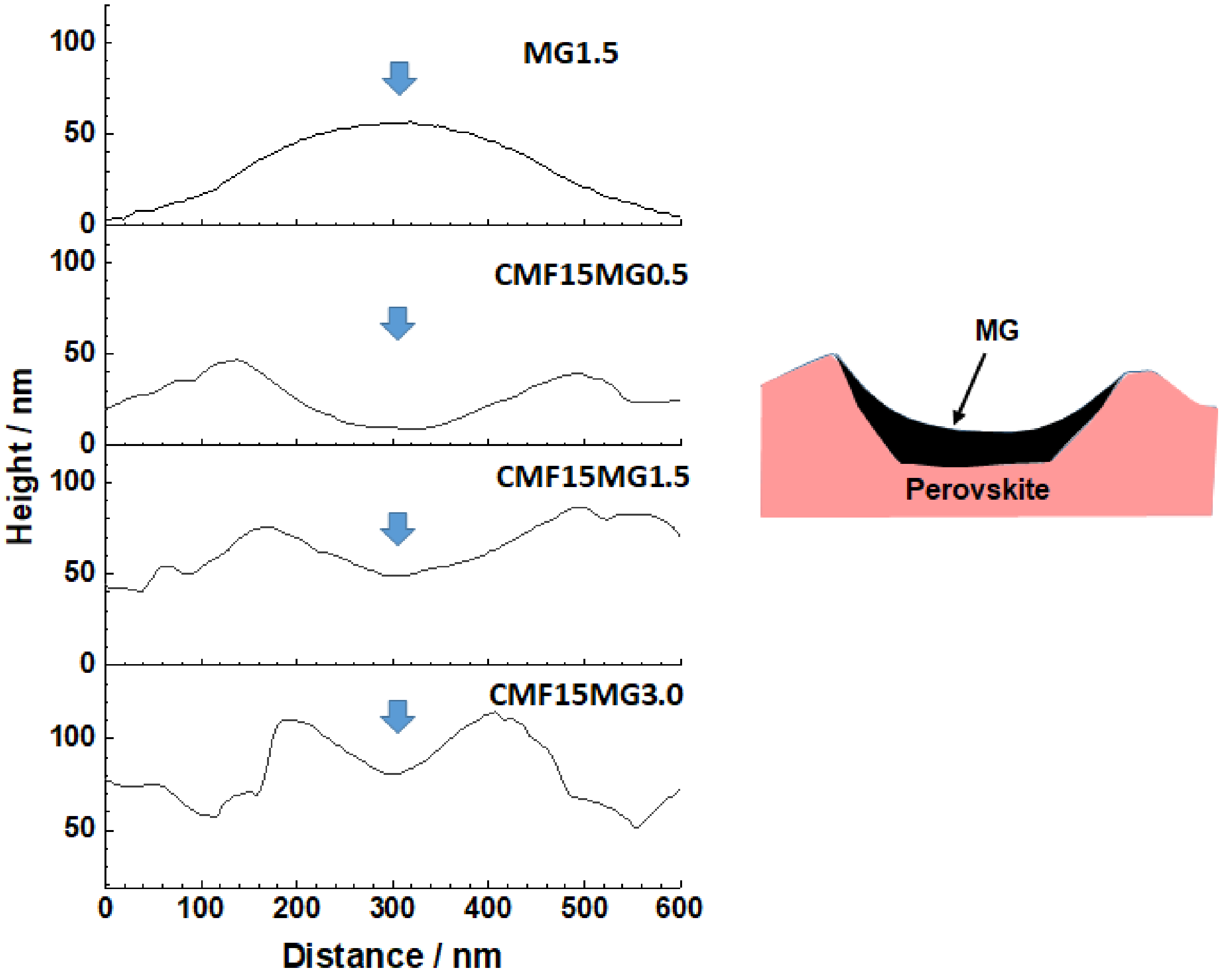

Fig. S4. Expanded AFM line profiles for the MGs highlighed from Fig. 2B. The x-axis distances have been shifted by constant values to place the MGs at the center of the x-axis range. The central positions of the MGs are indicated by the arrows. The cartoon depicts a collapsed MG within a perovskite pore. Support for this depiction comes from the expanded SEM view shown in Fig. S5.

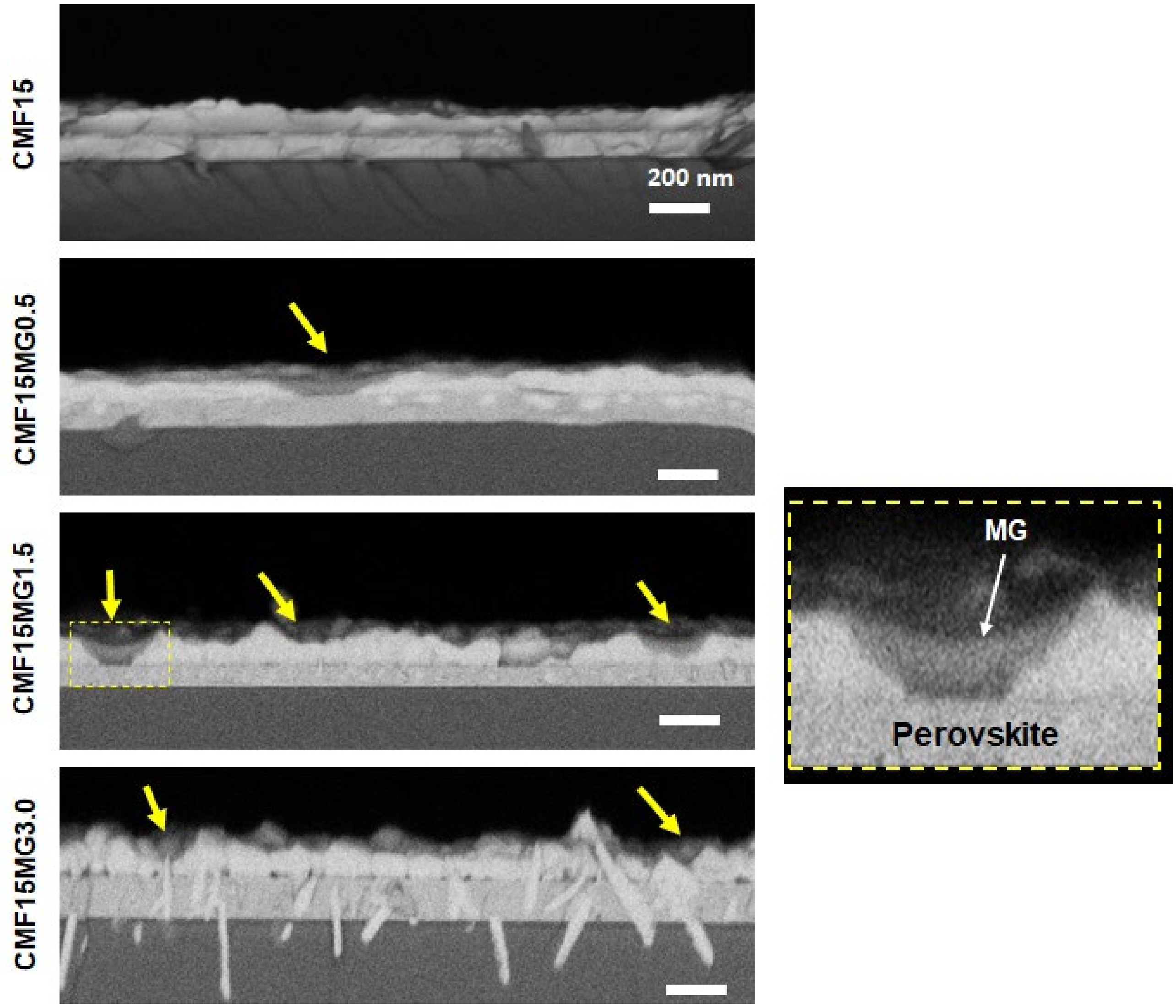

Fig. S5. Cross-section SEM images abtained using backscattered mode for various perovskite films. An expanded view of a MG in a pore of the CMF15MG1.5 film is also shown.

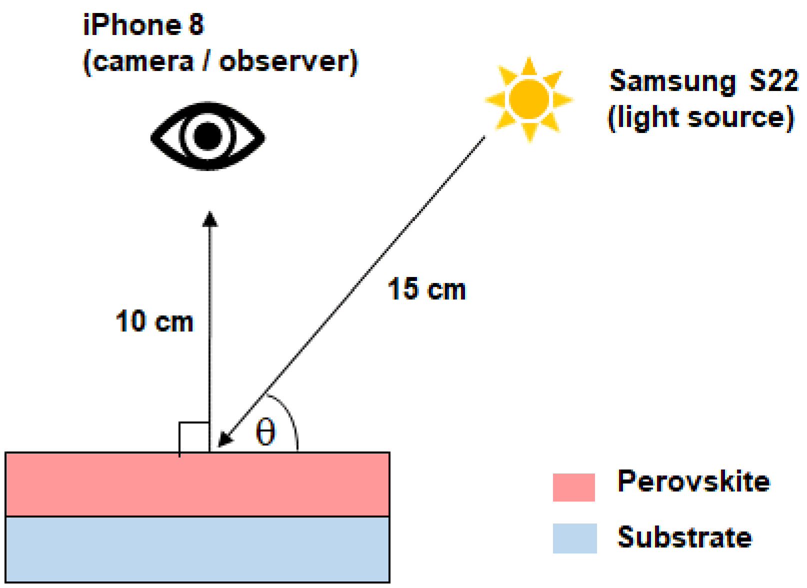

Fig. S6. Depiction of the arrangement for the reflection experiments.

Additional Note 1: Ray tracing for predicting colors of the films and devices

In the following a simple derivation is provided based on Bragg's law to predict the wavelength (λ), and hence color, of the perovskite films containing 2D MG arrays when the angle of light incident on the arrays (θ) is varied. For simplicity the MGs are considered as spheres with a different refractive index to that of the perovskite. The refractive index of the substrate is taken to be zero. Furthermore, the observer is normal to the surface and thin film interference is not considered. The separation between the MG centers is the period (*D*, Fig. S7). The path length difference for the two beams of light (= $m\lambda$, where m is an integer with unity being used here) is AB which is equal to $D\cos\theta$. Consequently, for the geometry used in our reflection experiments the following equation applies.

$$\lambda = Dcos\theta \qquad \text{(S1)}$$

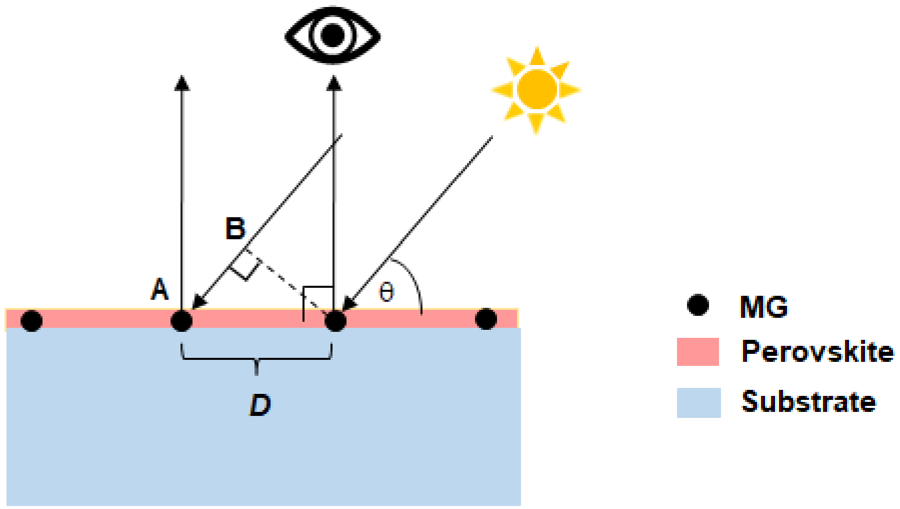

Fig. S7. Geometry and path length differences for non-close-packed 2D array of MGs deposited at a surface where the reflected light is perpendicular to the surface after being incident at an angle of θ.

The color observed is dependent on θ and *D*. The incident light is modulated via interference due to the coherent superposition of reflected electromagnetic waves generated at multiple MG-filled pores in the perovskite-MG hybrid films. We show in this study that tuning *D* via precursor composition produces aesthetically pleasing STPSCs.

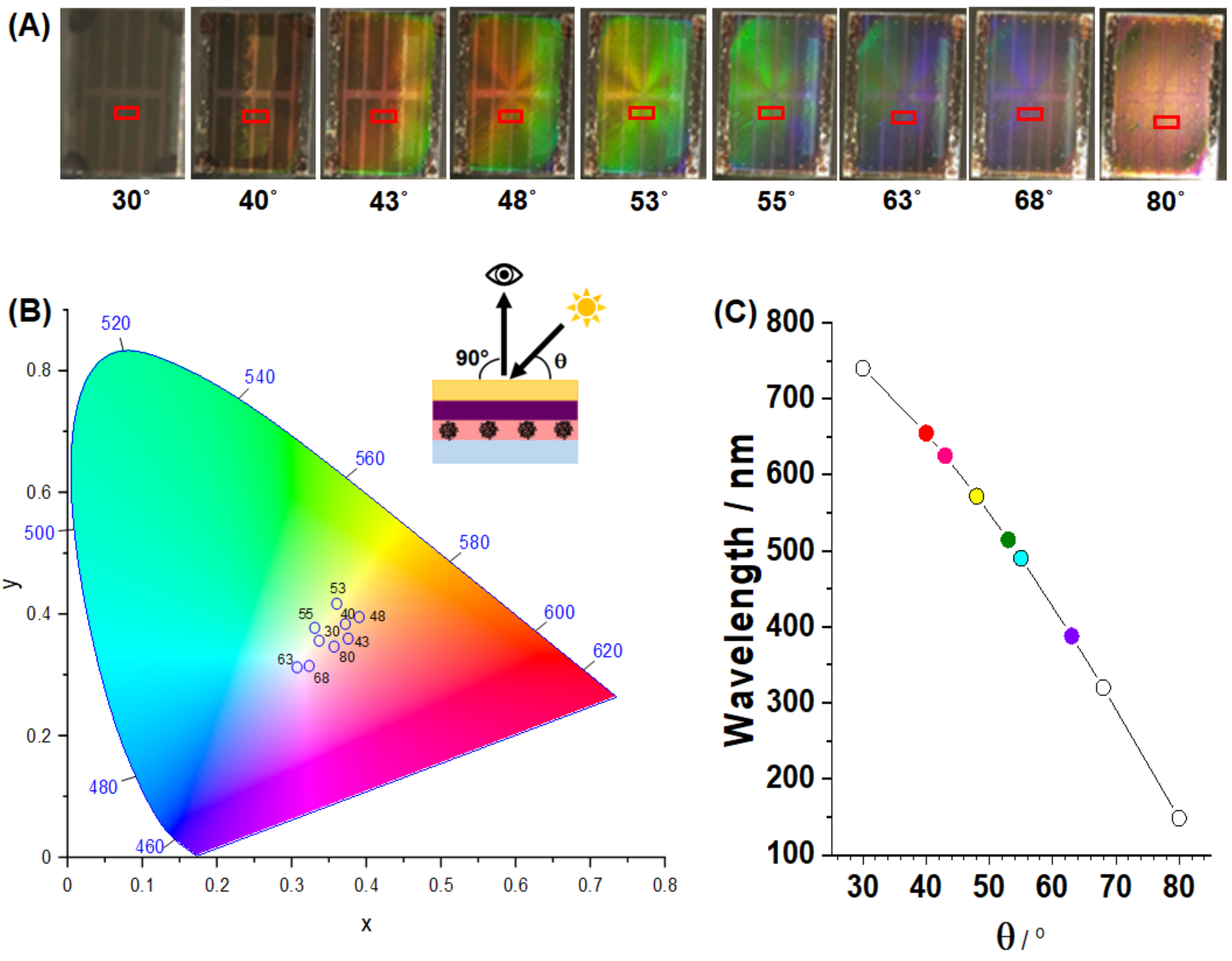

Fig. S8. (A) Photographs of CMF15MG1.5 devices showing the reflected light and the values of incident light angle (θ). The red rectangles show the regions sampled for CIE analysis. **(B)** *xy* color coordinates obtained from the sampled areas shown in (A). The geometry of the experiment is depicted. **(C)** The wavelengths of the reflected light for CMF15MG1.5 calculated using Equation (2) are shown for the θ values used in (A).

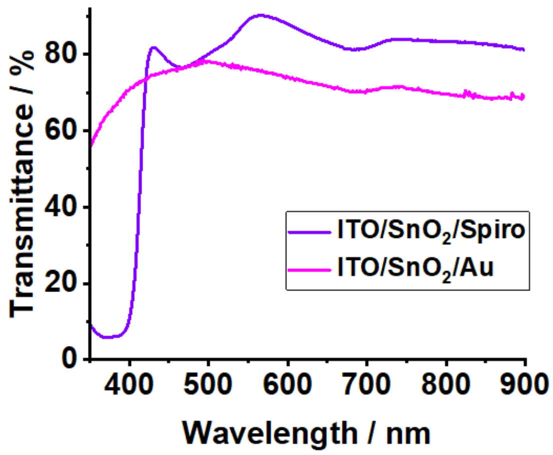

Fig. S9. UV-visible transmittance spectra for glass/ITO/SnO_2 coated with Spiro-OMeTAD or Au.

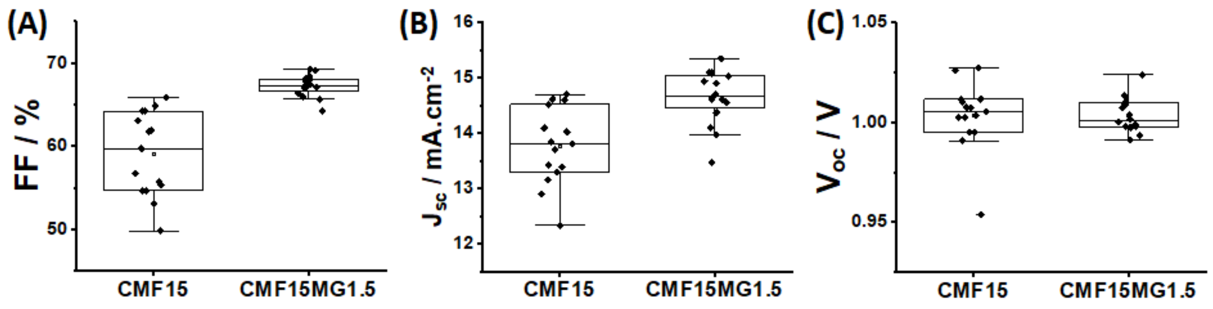

Fig. S10. Box plots for the CMF15 and CMF15MG1.5 devices showing the measured **(A)** fill factors, **(B)** short-circuit current densities and **(C)** open circuit voltage values.

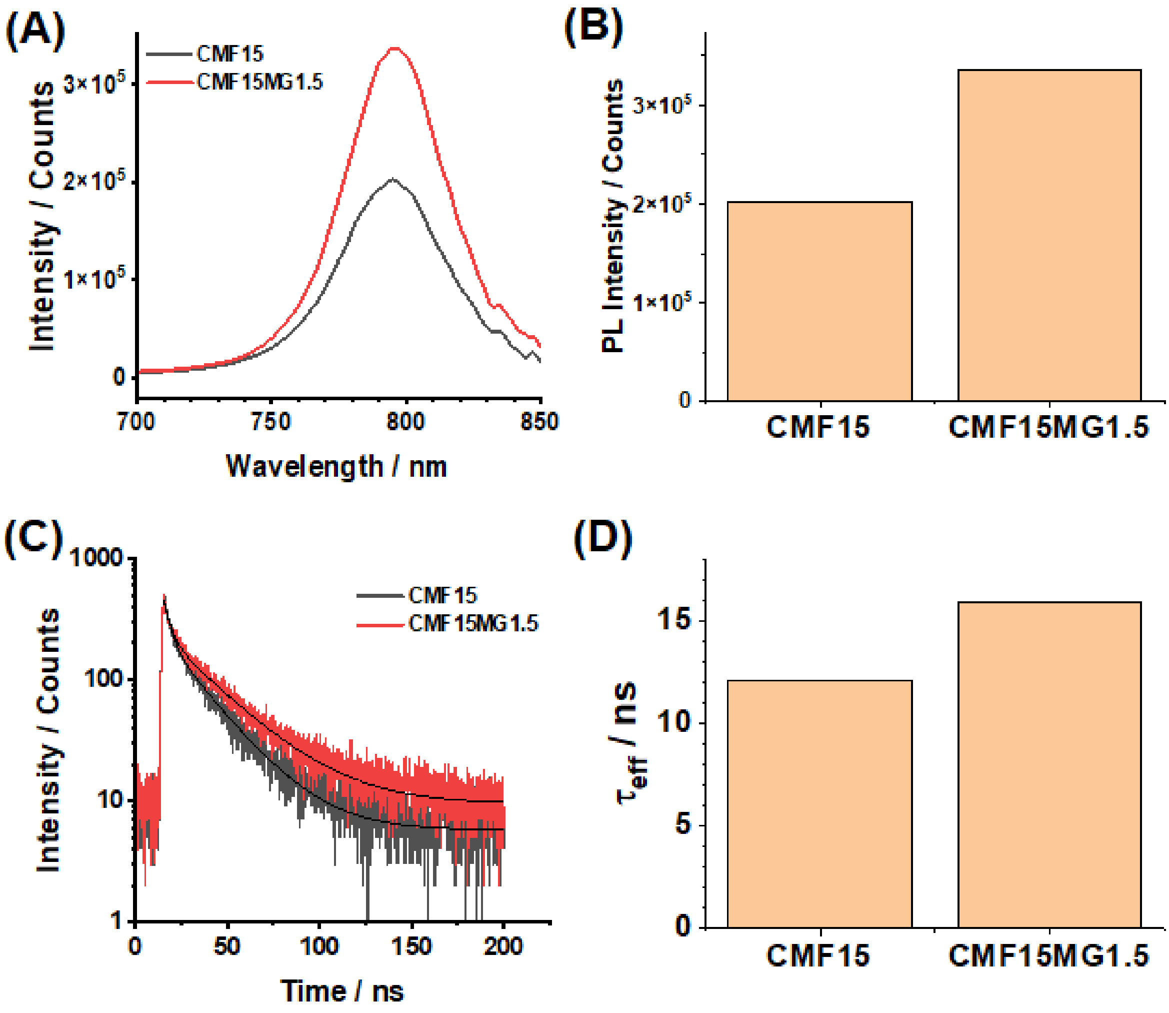

Fig. S11. (A) Steady-state PL spectra for the films deposited on glass/ITO/SnO_2. **(B)** Maximum PL intensities from (A). **(C)** Time-resolved PL decay curves. The data were fit using[6, 7] $I_{PL} = A\exp\left(-{}^{t}/_{\tau_{fast}}\right) + B\exp\left(-{}^{t}/_{\tau_{slow}}\right)$ where I_{PL} is the PL intensity, t is the decay time, A and B parameters are the relative amplitudes for the fast (τ_{fast}) and slow (τ_{slow}) lifetimes, respectively. **(D)** effective decay times from the fits to the data obtained in (C) using the equation[7] $\tau_{eff} = {(A\,\tau_{fast} + B\,\tau_{slow})}/_{(A+B)}$. The fitting parameters are shown in Table S2.

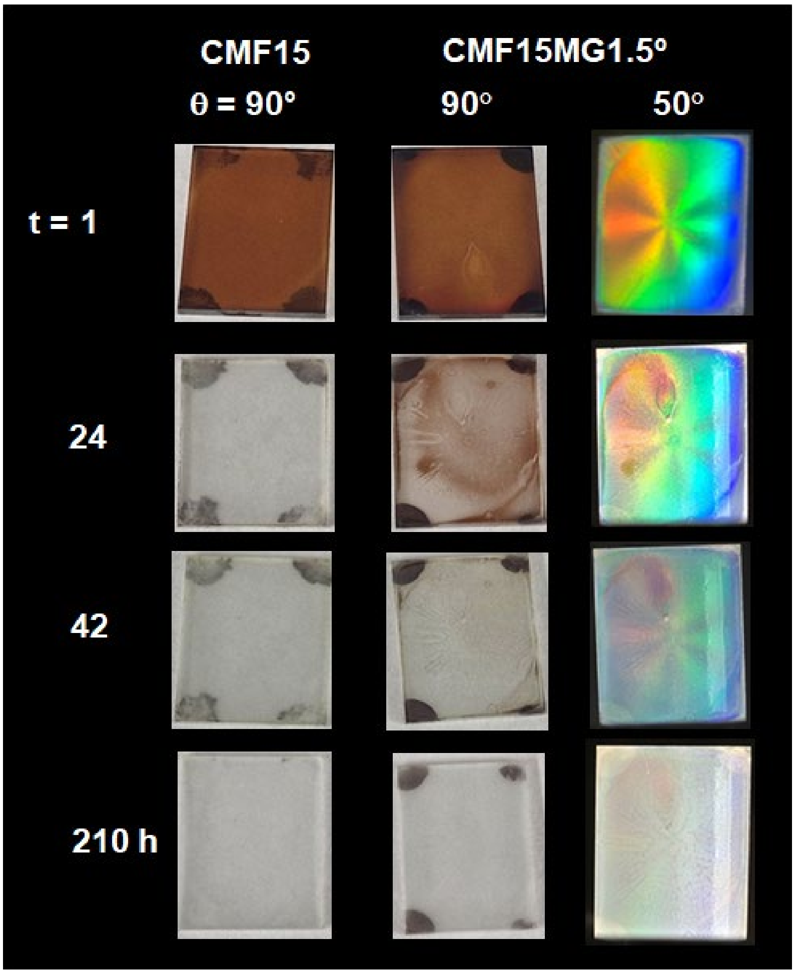

Fig. S12. Moisture stability at 90% RH and room temperature. The value for θ is the angle of the incident light. $\theta = 90°$ is the non-colored mode. The third column shows CMF15MG1.5 films using the colored mode ($\theta = 50°$).

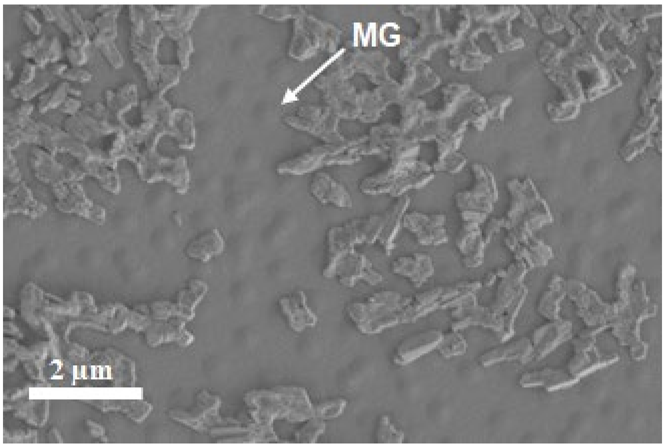

Fig. S13. SEM images for aged CMF15MG1.5 film (90% RH at room temperature) at 210 h. Compare this image with that of the original, non-degraded, CMF15MG1.5 film (Fig. 2A). A MG that was previously surrounded by perovskite is highlighted.

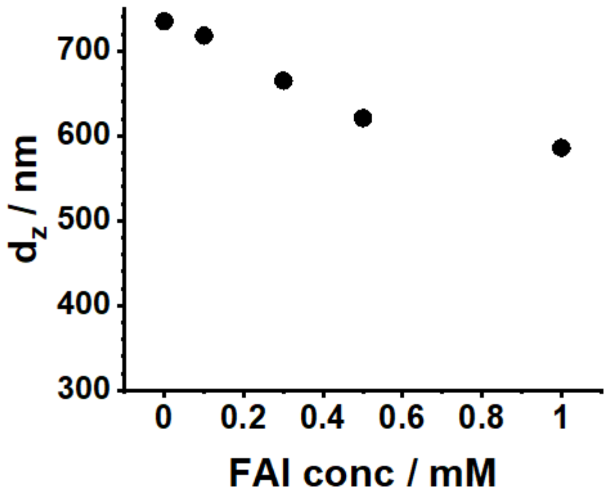

Fig. S14. Variation of z-average diameter (d_z) for the MG particles measured at 20 °C when dispersed in water containing different concentrations of FAI.

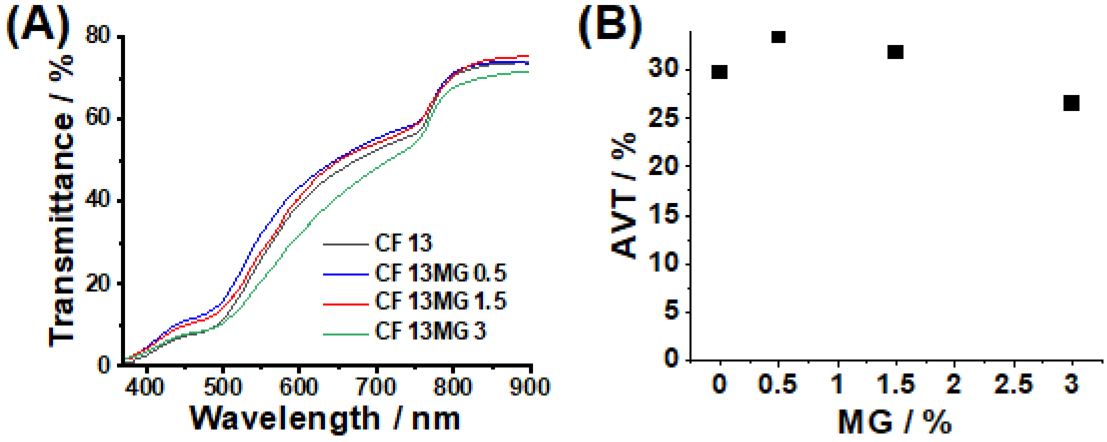

Fig. S15. (A) Transmittance spectra and **(B)** average visible transmittance data for CF13MGy films.

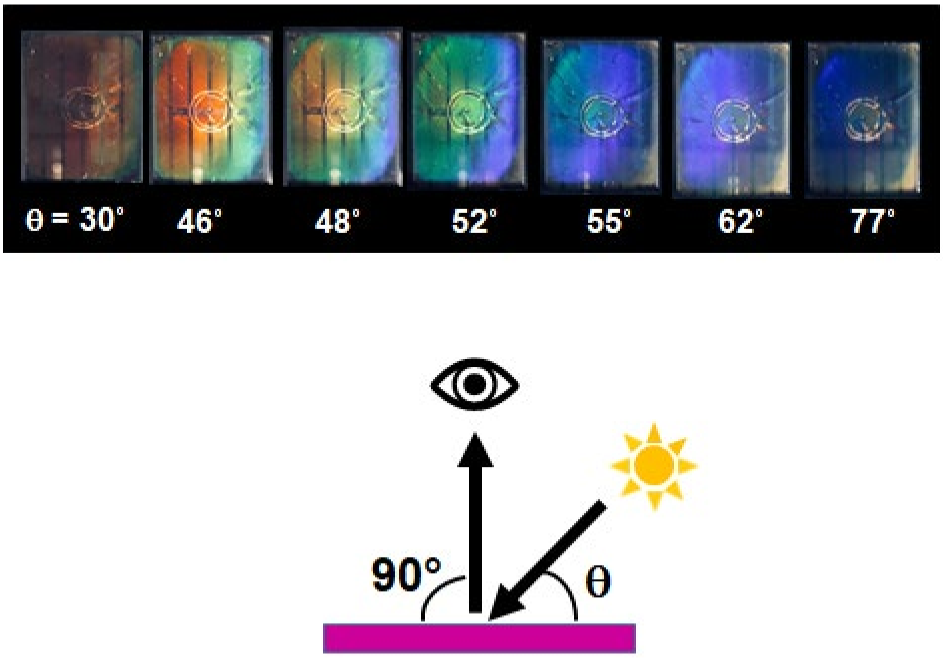

Fig. S16. Photographs of a CF13MG1.5 device obtained from the reflected light at various incident light angles. The geometry used for reflection is shown.

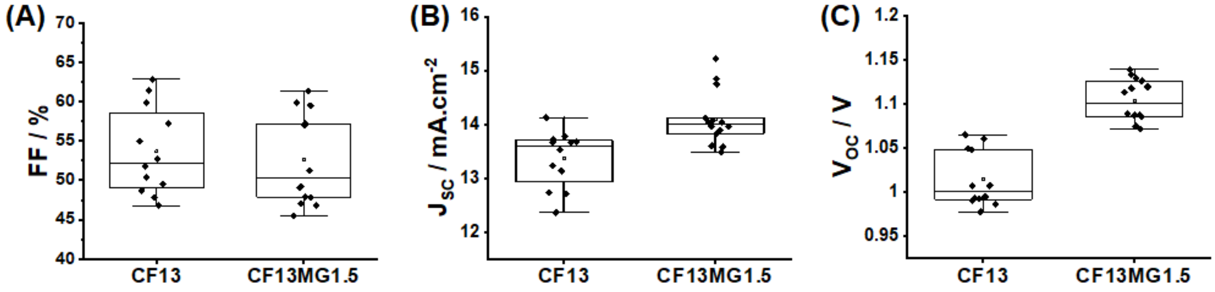

Fig. S17. Box plots of the CF13 and CF13MG1.5 devices showing the **(A)** FF, **(B)** J_{sc} and **(C)** V_{oc} data.

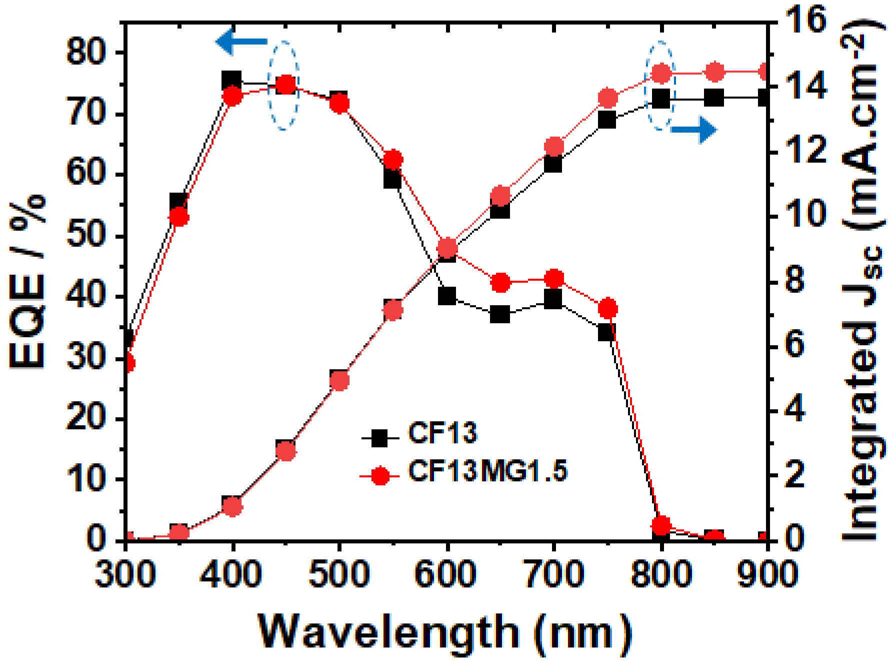

Fig. S18. EQE spectra for CF13 and CF13MG1.5 devices.

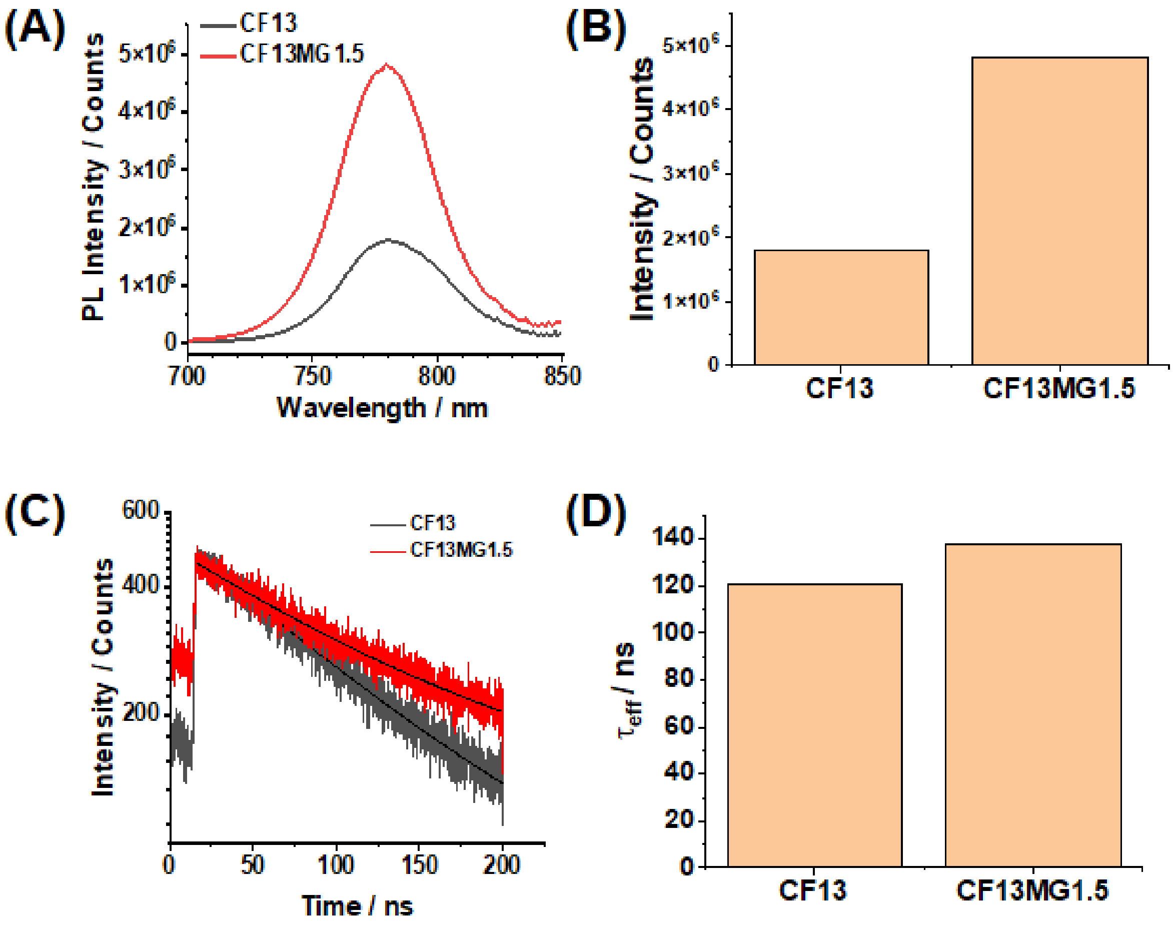

Fig. S19. (A) Steady-state PL spectra for the CF13MG1.5 and CF13 films deposited on glass/ITO/SnO_2. **(B)** Maximum PL intensities from (A). **(C)** Time-resolved PL decay curves of the CF13 and CF13MG1.5 films. The data were fit using $I_{PL} = B\exp\left(-{}^{t}/_{\tau_{slow}}\right)$ where I_{PL} is the PL intensity, t is the decay time, B parameters is the amplitude for the (τ_{slow}) lifetime. **(D)** effective decay times from the fits to the data obtained in (C). The fitting parameters are shown in Table S2.

Table S1. Performance summary of the double and triple-cation STPSCs.

System	Scan direction	V_{oc} (Volts)	J_{sc} (mA.cm^{-2})	FF (%)	PCE (%)
**CMF15**	Forward	1.00 ± 0.01	13.75 ± 0.70	56.53 ± 5.17	7.76 ± 0.78
	Reverse	1.00 ± 0.02	13.76 ± 0.67	59.04 ± 4.87	8.15 ± 0.75
	Average	1.00 ± 0.03	13.76 ± 0.68	57.79 ± 7.08	7.96 ± 1.12
	**Best**	**1.00**	**13.81**	**65.86**	**9.14**
**CMF15MG1.5**	Forward	1.00 ± 0.01	14.70 ± 0.48	65.32 ± 1.38	9.52 ± 0.38
	Reverse	1.00 ± 0.01	14.66 ± 0.47	67.26 ± 1.25	9.89 ± 0.36
	Average	1.00 ± 0.01	14.68 ± 0.47	66.29 ± 1.63	9.71 ± 0.41
	**Best**	**1.00**	**15.34**	**69.09**	**10.60**
**CF13**	Forward	0.97 ± 0.04	13.38 ± 0.50	46.28 ± 2.05	6.04 ± 0.59
	Reverse	1.01 ± 0.03	13.36 ± 0.40	53.66 ± 5.28	7.28 ± 0.79
	Average	0.99 ± 0.04	13.37 ± 0.50	49.97 ± 5.45	6.66 ± 0.93
	**Best**	**0.99**	**13.52**	**62.85**	**8.44**
**CF13MG1.5**	Forward	1.07 ± 0.03	14.13 ± 0.48	44.81 ± 4.71	6.79 ± 0.69
	Reverse	1.10 ± 0.02	14.10 ± 0.48	52.63 ± 5.51	8.18 ± 0.88
	Average	1.09 ± 0.03	14.11 ± 0.49	48.72 ± 6.45	7.49 ± 1.05
	**Best**	**1.09**	**15.22**	**57.11**	**9.45**

Table S2. Parameters of the fitting TRPL data for the films studied.

**System**	τ_{fast} **(ns)**	τ_{slow} **(ns)**	**A (Counts)**	**B (Counts)**	τ_{eff} **(ns)**
**CMF15**	3.49	22.38	249.94	208.86	12.09
**CMF15MG1.5**	3.52	28.45	221.80	220.34	15.94
**CF13**	-	121	-	430	121
**CF13MG1.5**	-	138	-	342	138

References

1. S. Zhou and B. Chu, *J. Phys. Chem. B*, 1998, **102**, 1364-1371.
2. Y. Min, X. Cao, H. Tian, J. Liu and L. Wang, *Chemistry*, 2021, **27**, 2065-2071.
3. C. Cai, W. Liu and X. Han, *Optik*, 2014, **125**, 1240-1242.
4. Pillow, https://pillow.readthedocs.io/en/stable/reference/Image.html, (accessed July, 2023).
5. Colour, https://colour.readthedocs.io/en/develop/index.html, (accessed July, 2023).
6. M. B. Faheem, B. Khan, C. Feng, S. B. Ahmed, J. Jiang, M. U. Rehman, W. S. Subhani, M. U. Farooq, J. Nie, M. M. Makhlouf and Q. Qiao, *ACS Appl Mater Interfaces*, 2022, **14**, 6894-6905.
7. P. Caprioglio, F. Zu, C. M. Wolff, J. A. Márquez Prieto, M. Stolterfoht, P. Becker, N. Koch, T. Unold, B. Rech, S. Albrecht and D. Neher, *Sustain. Energy Fuels*, 2019, **3**, 550-563.
